# High-mobility Group Box 1 Contributes to Hypoxic-Ischemic Brain Damage by Facilitating Imbalance of Microglial Polarization through RAGE-PI3K/Akt Pathway in Neonatal Rats

**DOI:** 10.7150/ijms.78641

**Published:** 2022-11-21

**Authors:** Yanyan Sun, Xing Zhu, Kaiyi Zhu, Jie Yu, Lin Cheng, Mingyan Hei

**Affiliations:** 1 Department of Hematology, Affiliated Cancer Hospital of Zhengzhou University, Henan Cancer Hospital, Zhengzhou, Henan, 450000, China.; 2 Department of Neonatology, Neonatal Center, Beijing Children's Hospital, Capital Medical University, Beijing, 100045, China.; 3 National Center for Child Health, Beijing, 100045, China.; 4 Key Laboratory of Major Diseases in Children, Ministry of Education, Beijing, 100045, China.

**Keywords:** Hypoxic-ischemic brain damage, HMGB1, Microglia, Polarization, Neuroinflammation

## Abstract

High mobility group box 1 (HMGB1) is a damage-associated molecular pattern integral for hypoxic-ischemic brain damage (HIBD) in neonatal rats since it regulates the phenotypic polarization of microglia, as depicted in our previous studies. Since this mechanism is not clear, this study establishes an oxygen-glucose deprivation (OGD) model of highly aggressively proliferating immortalized microglia while modulating the expression of HMGB1 by plasmid transfection. The M1/M2 microglial phenotype and receptor for advanced glycation end products-phosphoinositide 3-kinase/Akt (RAGE-PI3K/Akt) activation were evaluated, showing that HMGB1 promoted the polarization of microglia to the M1 phenotype under OGD conditions. Meanwhile, RAGE, which is the main receptor of HMGB1, was activated, and phosphorylation of PI3K/Akt was upregulated. However, knockdown or inhibition of HMGB1 can weaken the activation of RAGE and phosphorylation of PI3K/Akt. The inhibition of HMGB1 or RAGE-PI3K/Akt attenuated microglial polarization to the M1 phenotype and promoted M2 microglial polarization instead, reducing the release of pro-inflammatory factors. In the neonatal HIBD rat model, the RAGE-PI3K/Akt pathway was activated seven days after hypoxic-ischemic (HI) exposure, and the activation was partly inhibited after pretreatment with the HMGB1 inhibitor. Concurrently, inhibition of the HMGB1-RAGE-PI3K/Akt pathway alleviated neuronal damage in the hippocampus. These findings verified that HMGB1 could lead to an imbalance in M1/M2 microglial polarization through activation of the RAGE-PI3K/Akt signaling pathway under OGD conditions. Obstructing this pathway may attenuate the imbalanced polarization of microglia, enabling its utilization as a therapeutic strategy against brain injury in HIBD.

## 1. Introduction

High mobility group box 1 (HMGB1), a highly preserved nuclear DNA-binding protein, is a damage-associated molecular pattern that participates in many immune-related diseases of the central nervous system (CNS), including stroke, epilepsy, multiple sclerosis, and traumatic brain injury 1, 2. Our previous studies proved that in neonatal rats, the expression of HMGB1 significantly increased in the cerebral cortex after hypoxia-ischemia (HI) insult, and the M1/M2 microglial polarization in the cortex was imbalanced. While blockage of HMGB1 expression partially attenuated this imbalanced polarization, alleviating brain damage 3. These outcomes highlight that HMGB1 may generate an imbalanced M1/M2 microglial polarization and cause neuronal injury. However, the mechanism of this phenomenon is unclear and needs to be elucidated.

The receptor for advanced glycation end products (RAGE) is one of the main receptors of HMGB1 4. Its activation is related to inflammation, apoptosis, autophagy, proliferation and pathogenesis of nervous system diseases, such as Alzheimer's disease and peripheral neuropathy 5. RAGE expressed on microglia could mediate the neurotoxicity of HMGB1 in an animal model of ischemic stroke. Transplantation of bone marrow-derived cells into the brain after RAGE knockout can effectively reduce the area of cerebral infarction 6. A recent study exhibited that inflammation can be induced in microglia by the activation of HMGB1-RAGE. Furthermore, knockdown of HMGB1 expression through gene silencing can inhibit the activation of RAGE and alleviate neuroinflammation 7. Accordingly, we believe that HMGB1 may activate the downstream signaling pathway through the activation of RAGE in the HIBD model, which leads to the imbalanced polarization of M1/M2 microglia.

The phosphoinositide3-kinase/Akt (PI3K/Akt) pathway has a primary role in a variety of cell activities, including cell survival, proliferation, metabolism, neurological disease, and tumor growth 8, 9. PI3K is a lipid kinase characterized by its ability to phosphorylate the 3'-hydroxyl (3'-OH) group of inositol phospholipids in the plasma membrane 10. A downstream phosphorylated target of PI3K is serine-threonine kinase (Akt), which works closely with PI3K 11. Although the PI3K/Akt signaling pathway is deemed an anti-apoptotic pathway, studies denote that it can promote the production of inflammatory cytokines 12-14. In particular, PI3K is a key downstream protein of RAGE and can decrease the expression of pro-inflammatory factors in microglia if its pathway is inhibited 15. Previous studies have illustrated that PI3K/Akt signaling pathway was involved in M1/M2 polarization of macrophage/microglia 16. It is hypothesized that in the HIBD model, HMGB1 may facilitate the polarization of M1/M2 microglia through RAGE-PI3K/Akt pathway, leading to brain damage.

In this study, an OGD model of highly aggressively proliferating immortalized (HAPI) microglial cells and a neonatal rat model of HI were employed. In OGD cell model, the expression of HMGB1 was modulated by plasmid transfection. The polarization of M1/M2 microglia was detected by flow cytometry, quantitative polymerase chain reaction (qPCR) and enzyme-linked immunosorbent assay (ELISA), and the activation of the RAGE-PI3K pathway was evaluated via western blotting. In HIBD rat model, the HMGB1 inhibitor was utilized. The activation of RAGE-PI3K/Akt was detected by western blotting, and the neuronal damage was estimated by immunofluorescence. We found that HMGB1 participated in the imbalanced polarization of M1/M2 microglia by activating the RAGE-PI3K pathway, and inhibiting of HMGB1-RAGE-PI3K/Akt could alleviate neuronal damage in HIBD rat model.

## 2. Materials and Methods

### 2.1 Animals and Ethical Permission

Perinatal Sprague-Dawley (SD) rats were obtained from the Capital Medical University in Beijing, China. To minimize the risk of infection, the rats were housed in laminar flow hoods and pathogen-free rooms. All animal experiments were approved by the ethics committee of the Capital Medical University. Efforts were made to minimize the number of animals and mitigate their suffering during the experiment.

### 2.2 HIBD Rat Model and Drug Administration

Postpartum day seven (P7) rat pups were divided randomly into three groups, namely Sham+phosphate-buffered saline (PBS), HI+PBS, and HI+Glycyrrhizin (GL). In the HI+GL group, GL was administered 1 h before artery ligation (20 mg/kg, i.p.). In the other two groups, an equal volume of 0.01M PBS was administered. Subsequently, minor modifications were introduced to Rice and Vannucci's HIBD model. Isoflurane was used to anesthetize the rat pups, and their left common carotid artery was permanently ligated. Pups were then returned to the dam for a two-hour recovery period before the initiation of two-hour hypoxia exposure (37 °C, 8% O_2_/92% N_2_). The sham-operated control animals were given only a small incision on the left side of the neck, and the left common carotid artery was isolated without artery ligation or hypoxia treatment.

### 2.3 HAPI Microglia Cultures, Treatment and Oxygen-glucose Deprivation

Cells from the microglia-like cell line HAPI were seeded into 6-well plates at 1×10^5^/mL and incubated overnight in a high-glucose Dulbecco's Modified Eagle medium (DMEM) containing 10% fetal bovine serum (FBS). To explore the mechanism of HMGB1 affecting microglial polarization, the inhibitors of HMGB1 (GL, 55 μM), RAGE (FPS-ZM1,1.2 μM), PI3K (BEZ235, 25 μM), Akt (AZD5363, 2.5 μM) and recombinant HMGB1 (r-HMGB1, 10 ng/mL) were used. All the above inhibitors were purchased from Selleck (Shanghai, China), and r-HMGB1 was synthesized by CUSABIO (Wuhan, China). After incubation for 2 hours, an oxygen-glucose deprivation (OGD) model was established to mimic the *in vivo* HI process. Briefly, the cells' medium was then replaced with glucose-free Earle's balanced salt solution, and they were placed in an oxygen-deprived incubator (93% N_2_/5% CO_2_/2% O_2_) at 37 °C for 6 hours.

### 2.4 Vector Construction and Cell Transfection

Using pUC57-rat-HMGB1 (GenScript) as a template, full-length complementary DNA (cDNA) of rat HMGB1 was amplified through PCR and then inserted into the XhoI/HindIIIsite of pcDNA3.1 to obtain pcDNA3.1-rat-HMGB1. To generate the small hairpin RNA (shRNA) plasmid targeting rat-HMGB1, the GAC CAT GTC TGC TAA AGAA sequences were cloned into pG2.2 to produce pG2.2-shRNA-rat-HMGB1. TTC TCC GAA CGT GTC ACGT sequences were designed to obtain a negative control shRNA. Next, HAPI microglia cells were seeded in six-well plates at the density of 5×10^5^/well and serum-free medium was changed 2 hours before transfection in the following day. Then, mixture of vectors and liposomes were prepared and transferred into HAPI cells using Lipofectamine^TM^ 2000 (Invitrogen). Briefly, vectors (4 μg) and Lipofectamine^TM^ 2000 (5 μL) were diluted with 100 μL serum-free opti-MEM (GIBCO), respectively, and left at room temperature for 5 minutes. These were then mixed into transfection complexes and left at room temperature for 20 minutes. Finally, 200 μL of the mixture was added to each well and the cells were cultured in an incubator (37 °C, 5% CO_2_). After 4 hours, the mixture was aspirated and DMEM with 20% FBS was added to each well. The transfection efficiency was evaluated by PCR and western blotting after 24 hours.

### 2.5 Enzyme-Linked Immunosorbent Assay (ELISA)

The culture supernatant from HAPI microglia was collected. Afterward, the samples were centrifuged at 3000 rpm for 10 minutes at room temperature (temperature of 20-25 °C) for the enzyme-linked immunosorbent assay(ELISA). The concentrations of TNF-α (Thermo Fisher, United States, 88-7340-22), IL-1β (R&D Systems, United States, DY501-05), and IL-10 (Abcam, United States, ab218796) were determined using respective ELISA kits, and all measurements were performed according to the manufacturer's protocols.

### 2.6 Reverse Transcription and qRT-PCR

Cells were collected, and total RNA was isolated using the TRIzol reagent (Invitrogen, United States). According to the manufacturer's protocol, the first-strand cDNA was synthesized using the Reverse Transcription System (Toyobo, Osaka, Japan). The relative expression level of messenger RNA (mRNA) was then evaluated using the SYBR Green Real-time PCR Master Mix Kit (Toyobo, Osaka, Japan) and quantified utilizing the Mastercycler^R^ep realplex qRT-PCR system (Eppendorf, Germany), with β-actin as the reference gene. All primers employed in the qRT-PCR reactions were procured from Sangon Biotech (Shanghai, China). The sequences of the primer pairs are described as follows: TNF-α (forward: 5'-CCG ATT TGC CAT TTC ATA CCAG-3'; reverse: 5'-TCA CAG AGC AAT GAC TCC AAAG-3'); IL-1β (forward: 5'-CTT CAA ATC TCA CAG CAG CAT-3'; reverse: 5'-CAG GTC GTC ATC ATC CCAC-3'); iNOS (forward: 5'-AAG GGA TCT TGG AGC GAG TT-3'; reverse: 5'-GAG GGG TAG TGA TGT CCA GG-3'); arginase-1 (forward: 5'-GCC CAT TCA CCT GAG TTT TGA-3'; reverse: 5'-ATT ACC TTC CCG TTT CGT TCC-3'); IL-4 (forward: 5'-CCC CAG AAT GCC TTG GTC TA-3'; reverse: 5'-TTC ATC ATA GCA ACA GCC GC-3'); β -actin (forward: 5'- CAC GAT GGA GGG GCC GGA CTC ATC -3' ; reverse: 5'- TAA AGA CCT CTA TGC CAA CAC AGT -3'). Finally, relative mRNA expression was calculated using the 2^-△△Ct^ method.

### 2.7 Flow Cytometry

Cells were harvested at a density of 1×10^5^ cells/well. Thereafter, the cells were stained with anti-CD86 (PE-conjugated, BD Bioscience) and anti-CD206 (PE-conjugated, Santa Cruz) antibodies and then incubated for 15 minutes on ice in the dark. Excess antibody was washed away in 1mL 0.01M PBS by centrifuging at 1500 rpm for 5 minutes. Lastly, the cells were analyzed using a FACS-Calibur flow cytometer (BD, USA) and Divasoftware (BD). In each case, isotypic control was implemented, and an appropriate isotype-matched control antibody was purchased from BD Bioscience.

### 2.8 Immunofluorescence Staining

Animals were anesthetized and transcardially perfused with 0.01M PBS and 4% paraformaldehyde (PFA) seven days after HI. The brains were immediately removed and post-fixed in 4% PFA. After dehydration with a sucrose gradient, 20 serial coronal sections were cut across the middle hemisphere. Sections were then washed thrice with 0.01M PBS, blocked with 5% bovine serum albumin (BSA), and used for active neuronal nuclei (NeuN) and microtubule association protein-2 (MAP2) staining. Eventually, the sections were incubated overnight at 4 °C with anti-NeuN (1:300 dilution, Abcam, ab177487) and anti-MAP2 (1:200 dilution, Proteintech,17490-1-AP) primary antibody. After three washes in 0.01M PBS, the sections were incubated with Cy3-conjugated goat anti-rabbit immunoglobulin G (IgG) (1:2000 dilution, Boster Biological Technology, BA1032) for one hour at room temperature. After three washes in 0.01M PBS, they were finally covered with diamidino-2-phenylindole (DAPI, 1:1000, Beyotime, C1002) for five minutes. For each staining, five non-overlapping digital microscopic images of the hippocampal areas were captured randomly using a fluorescence microscope (IX71, OLYMPUS, Japan), and the number of positive cells in the hippocampal CA1 area was distinguished via Image-Pro Plus 6.0.

### 2.9 Western Blotting

Western blotting was utilized to evaluate the expression of HMGB1, RAGE, PI3K, p-PI3K, Akt, p-Akt, and β-actin in HAPI microglia and in brain tissue. Frozen hippocampal samples and microglia cells were completely homogenized in lysis buffer containing phenylmethanesulfonyl fluoride, a phosphatase inhibitor, and radioimmunoprecipitation assay and centrifuged at 12,000 rpm for 15minutes at 4 °C. The supernatant was collected, together with the total protein extracted from the tissue. A BCA protein assay kit was utilized according to the manufacturer's instructions to determine the quantity of protein in the samples. Samples were separated by 10%-12% sodium dodecyl sulfate-polyacrylamide gel electrophoresis (SDS-PAGE) and transferred to polyvinylidene fluoride (PVDF) membranes. Membranes were blocked with 5% bovine serum albumin for 2 hours at room temperature and then incubated at 4 °C overnight with the following primary antibodies: rabbit anti-HMGB1(Abcam, ab18256), rabbit anti-RAGE (Abcam, ab3611), rabbit anti-PI 3kinasep85 alpha (Abcam, ab191606), rabbit anti- PI 3 kinasep85 alpha (phosphorY607) (Abcam, ab182651), rabbit anti-Akt (Proteintech, 10176-2-AP), rabbit anti-Akt (phosphor-S473) (Proteintech, 66444-1-ap), and rabbit anti-β-actin (Proteintech, 14395-1-AP). After three washes in TBST (0.01M TBS containing 0.1% Tween-20, the membranes were incubated with secondary antibodies (goat anti-rabbit IgG, IRDyeR 800CW Conjugated, 1:5000 dilution) at room temperature for two hours. Lastly, blotted protein bands were visualized using an infrared laser imaging system (Odyssey CLx, LI-COR, United States) and quantified through densitometry. The relative expression levels of the protein were normalized by the ratio of the target protein to β-actin.

### 2.10 Statistical Analysis

All data were illustrated as mean±SD and inspected using one-way analysis of variance (ANOVA) and Tukey's test for post hoc comparisons. Statistical Package for the Social Sciences 19.0 (SPSS, IBM, United States) and GraphPad Prism 5.0 (GraphPad, San Diego, California) were used accordingly, and statistical significance was set at p< 0.05.

## 3. Results

### 3.1 Effect of HMGB1 on the Polarization of Microglia under OGD Conditions

**Figure 1:**
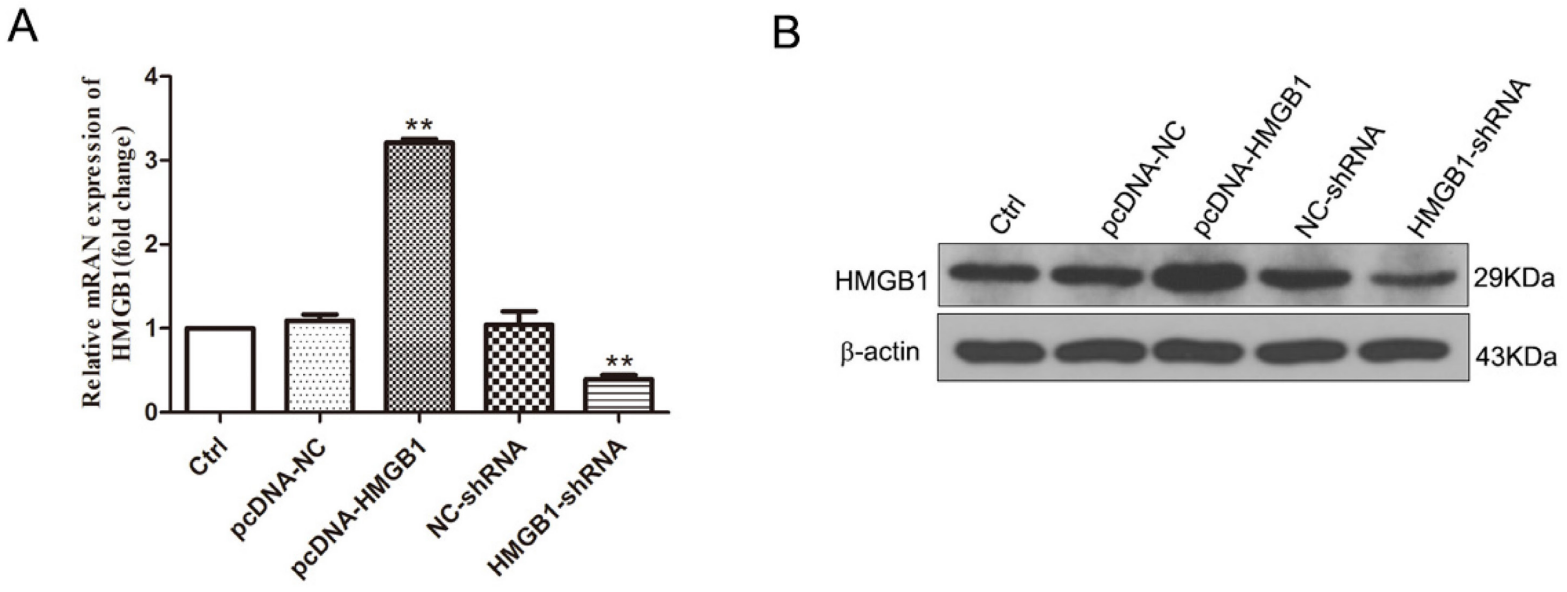
** The expression of HMGB1 modulated by cell transfection. A.** The mRNA expression of HMGB1 evaluated by q-PCR. Compared with the Ctrl group, the expression of HMGB1 in the pcDNA-HMGB1 group was significantly upregulated, and it was down-regulated in the HMGB1-shRNA group (n=3, ** p<0.01). **B.** The immunoblotting of HMGB1 detected by Western blot. Compared with the Ctrl group, the blotting in the pcDNA-HMGB1 group was enhanced, and it was weakened in the HMGB1-shRNA group.

Our previous study found that HMGB1 promoted the expression of M1-like cytokines and aggravates the neurotoxicity induced by microglia under OGD conditions. In this study, cell transfection technology was employed to enhance or downregulate the expression of HMGB1 to further explore the influence of HMGB1 on the polarization of M1/M2 microglia, dividing HAPI microglial cells into the following five groups: Ctrl, pcDNA-NC, pcDNA-HMGB1, NC-shRNA, and HMGB1-shRNA. Westernblotting and q-PCR were used to detect the expression of HMGB1 24 hours after transfection. The findings validate that the expression of HMGB1 in the pcDNA-HMGB1 group was significantly upregulated but significantly downregulated in the HMGB1-shRNA group (Fig. 1A, B), outlining that the cells were successfully transfected.

**Figure 2:**
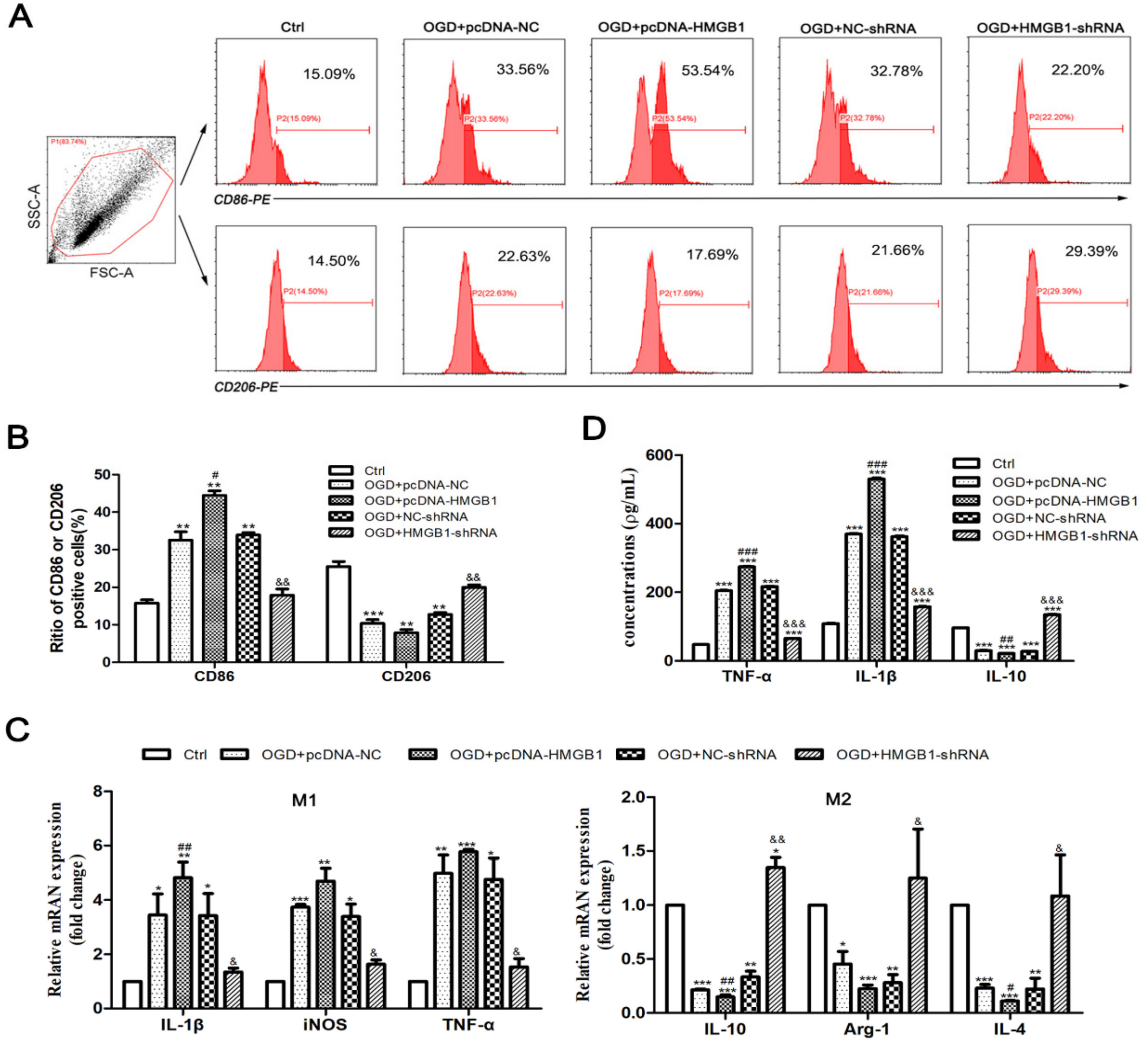
** The polarization of M1/M2 microglia after OGD treatment. A.** The phenotype of M1 and M2 microglia detected by flow cytometry. CD86-PE and CD206-PE represent the phenotype and fluorescence channel of M1 and M2 microglia, respectively. **B.** The percentage of CD86+ and CD206+ cells. **C.** The mRNA expression of M1 and M2 inflammatory factors. Compared with the Ctrl group, the M1 cytokines (IL-1β, iNOS and TNF-α) were up-regulated and the M2 cytokines (IL-10, Arginase1 and TGF-β) were down-regulated significantly in the OGD+pcDNA-NC group. In the OGD+pcDNA-HMGB1 group, the expression of M1 cytokine was further up-regulated, and the expression of M2 cytokine was down-regulated compared with the OGD+pcDNA-NC group. In the OGD+HMGB1-shRNA group, the expression trend of M1 and M2 cytokines was opposite to that of OGD+pcDNA-HMGB1 group. **D.** Expression of inflammatory factors in the supernatant detected by ELISA. After OGD treatment, the expression of TNF-α and IL-1β was up-regulated and the expression of IL-10 was down- regulated. After enhancing the expression of HMGB1 gene, the expression of TNF-α and IL-1β was further up-regulated, and the expression of IL-10 was further down-regulated, while silencing the HMGB1 gene could neutralize the above changes. N=3 per group. * Represents statistical significance compared with the Ctrl group, * p<0.05, ** p<0.01, *** p<0.001. #Represents statistical significance compared with the OGD+pcDNA-NC group, #p<0.05, ## p<0.01, ### p<0.001, '&' represents statistical significance compared with the OGD+NC-shRNA group, &p<0.05, &&p<0.01, &&&p<0.001.

Then cells were divided into the following five groups: Ctrl, OGD+pcDNA-NC, OGD+pcDNA-HMGB1, OGD+NC-shRNA, and OGD+HMGB1-shRNA. Flowcytometry was used to detect the phenotype of microglia (M1: CD86+, M2: CD206+), qPCR was utilized to determine the mRNA expression of cytokines, and ELISA was employed to discern the concentration of inflammatory factors in the culture supernatant. The outcomes of flow cytometry specified that the proportion of CD86+ cells significantly increased, while the proportion of CD206+ cells significantly reduced after the expression of the HMGB1 gene (OGD+pcDNA-HMGB1) was enhanced. When the HMGB1 gene (OGD+HMGB1-shRNA) was silenced, the proportion of CD86+ and CD206+ cells decreased and increased, respectively (Fig. 2A, B). The findings of the qPCR revealed that after the OGD treatment, the expression of M1 type cytokines (i.e., IL-1β, iNOS, and TNF-α) was upregulated to varying degrees (p<0.05), while the M2 type cytokines (i.e., IL-10, Arg-1, and IL-4) were downregulated (p<0.05). Further observation signified that the expression levels of IL-1β, iNOS, and TNF-α were further increased, while the expression levels of IL-10, Arg-1, and IL-4 were further decreased in the OGD + pcDNA-HMGB1 group. In the OGD+HMGB1-shRNA group, the expressions of IL-1β, iNOS, and TNF-α were downregulated, and the expressions of IL-10, Arg-1, and IL-4 were upregulated (Fig. 2C). Similar results were observed by ELISA (Fig. 2D). These results indicate that HMGB1 promoted M1 microglial polarization under OGD conditions.

### 3.2 RAGE-PI3K Signaling Pathway Activated under OGD Conditions

**Figure 3:**
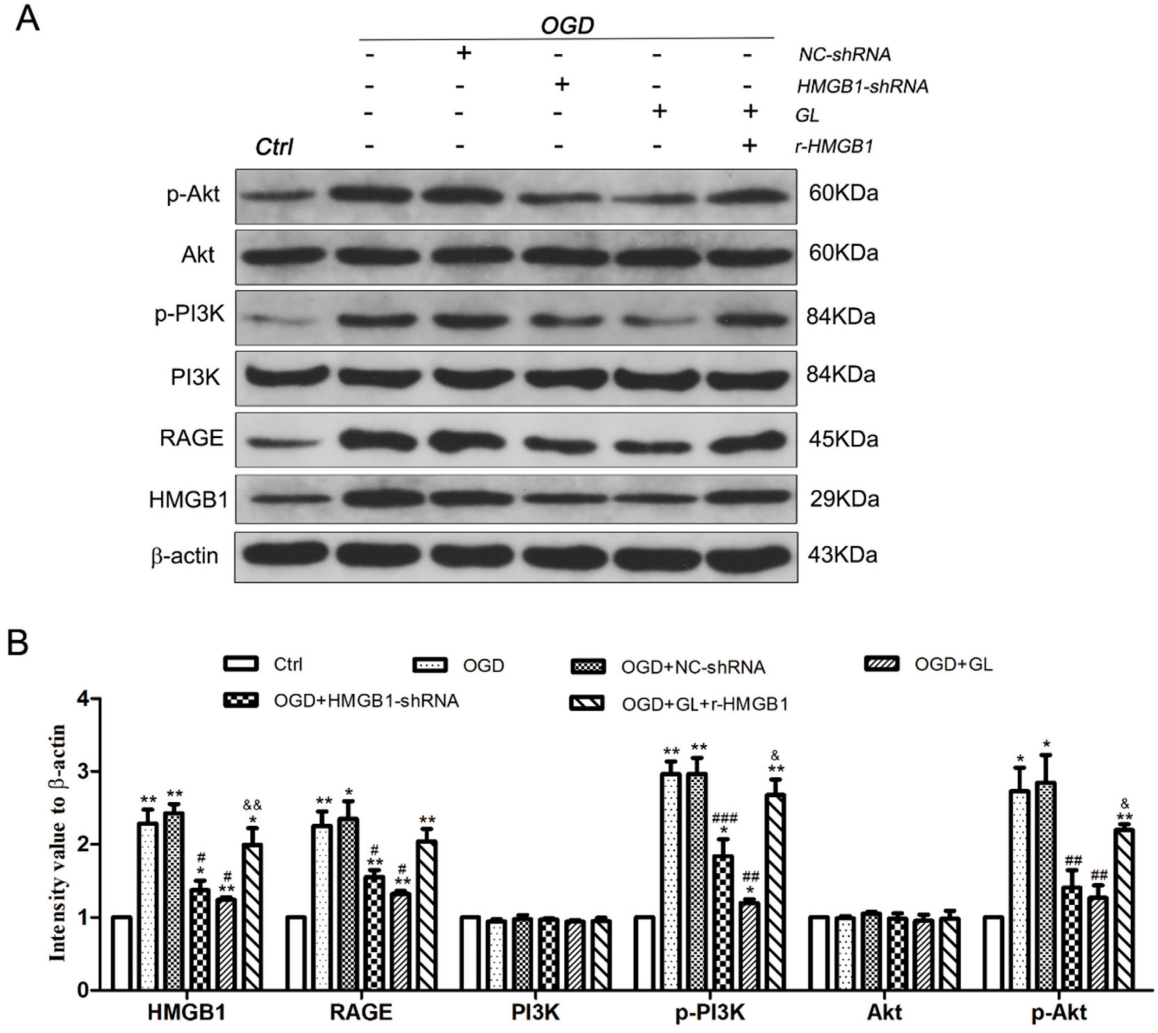
** Activation of RAGE-PI3K pathway under OGD conditions. A.** The immunoblots of HMGB1, RAGE, PI3K/p-PI3K and Akt/p-Akt. Compared with the Ctrl group, the expression of RAGE in the OGD group was upregulated, and the phosphorylation of P85 subunit and S473 occurred in PI3K-Akt pathway. **B.** Quantitative results of each protein. N=3 per group. * Represents statistical significance compared with the Ctrl group. * p<0.05, ** p<0.01, *** p<0.001. #Represents statistical significance compared with the OGD group. # p<0.05, ## p<0.01, ### p<0.001. '&' Represents statistical significance compared with the OGD+NC-shRNA group. &p<0.05, &&p<0.01, &&&p<0.001.

To further investigate the mechanism of the effect of HMGB1 on the M1/M2 polarization of microglia, western blotting was utilized to detect the expression of the RAGE-PI3K signaling pathway under different conditions. The results denoted that the expression of HMGB1 and RAGE in the OGD group was upregulated compared to the control group. Besides, phosphorylation of PI3K and Akt was activated. This notion indicated that the RAGE-PI3K pathway was activated under OGD conditions. Further analysis depicted that the expression of RAGE was downregulated and the phosphorylation of PI3K and Akt deteriorated after the expression of HMGB1 was inhibited. After the use of r-HMGB1, the expression of RAGE was enhanced, and the phosphorylation of PI3K and Akt was significantly upregulated. These outcomes demonstrated that the RAGE-PI3K/Akt pathway was activated under OGD conditions, and phosphorylation and activation of RAGE-PI3K/Akt were dependent on the regulation of HMGB1 (Fig. 3A and B).

### 3.3 Effect of Inhibiting RAGE-PI3K on the Polarization of M1/M2 Microglia

**Figure 4:**
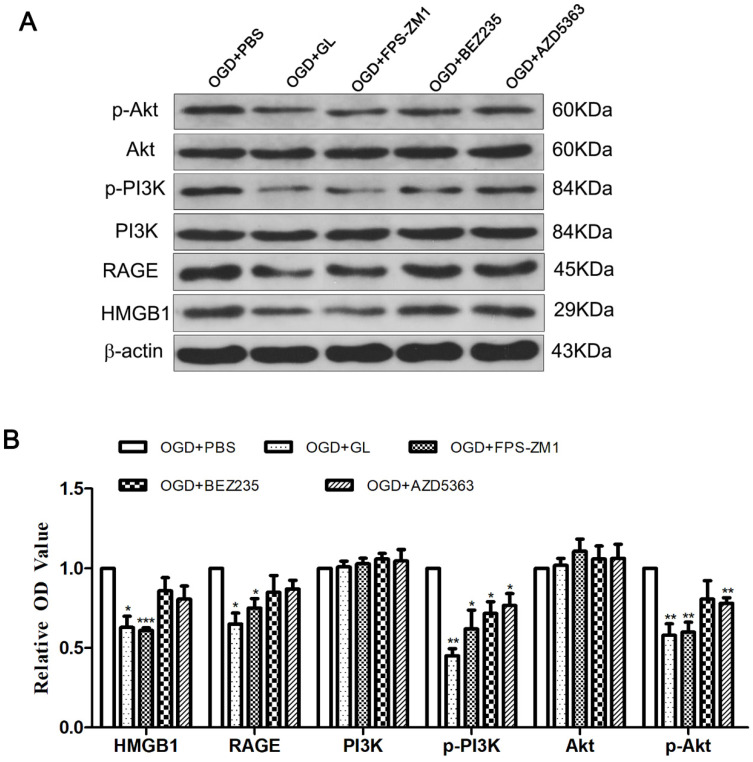
** The expression of related proteins after inhibiting HMGB1 and RAGE-PI3K pathway. A.** The immunoblots of HMGB1, RAGE, PI3K/p-PI3K and Akt/p-Akt after inhibiting HMGB1, RAGE or PI3K/Akt. **B.** Quantitative results of each protein. Compared with the OGD+PBS group, the expression of p-PI3K and p-Akt was weakened in the OGD+GL and OGD+FPS-ZM1 group, and the expression of HMGB1 and RAGE in the OGD+BEZ235 group and OGD+AZD5363 group did not change significantly. N=3 per group. * Represents statistical significance compared with the OGD+PBS group. * p<0.05, ** p<0.01, *** p<0.001.

HMGB1 inhibitors and related inhibitors of the RAGE-PI3K pathway were applied to further clarify the role of this pathway in regulating HMGB1 in terms of microglial polarization. Western blotting was performed to ascertain the expression of each protein after treatment with inhibitors. Notably, the HMGB1 inhibitor GL effectively constrained the expression of RAGE, p-PI3K, and p-Akt. Meanwhile, the RAGE inhibitor FPS-ZM1 could effectively hinder the expression of p-PI3K and p-Akt, implying that HMGB1-RAGE is imperative for the phosphorylation of PI3K-Akt. The inhibition of the PI3K-Akt pathway by BEZ235 and AZD5363 reduced the expression of p-PI3K and p-Akt, but the expression of HMGB1 and RAGE did not change significantly. These outcomes suggest that the PI3K-Akt pathway is downstream of HMGB1 and RAGE (Fig. 4A and B).

**Figure 5:**
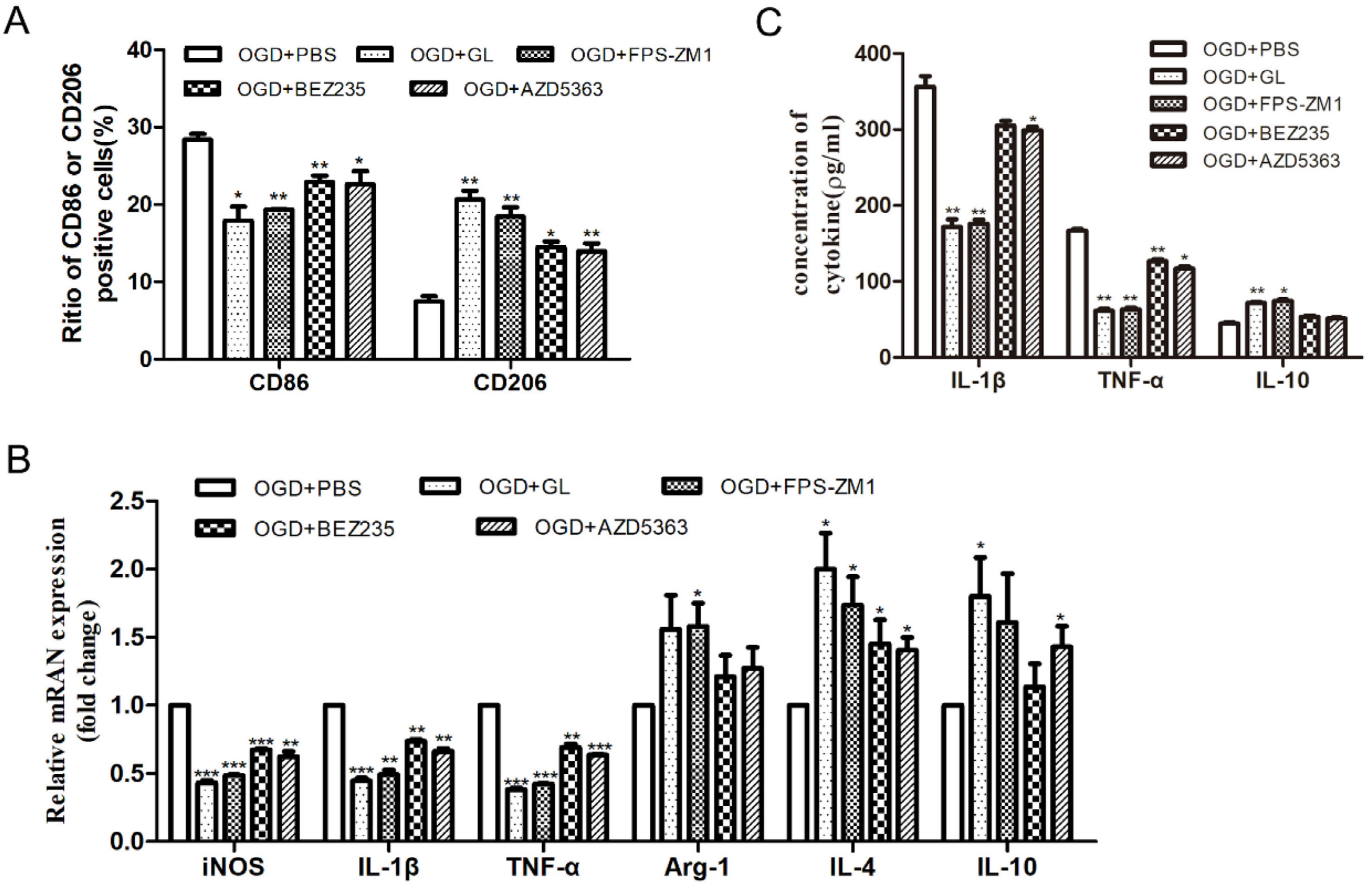
** The effect of inhibiting HMGB1/RAGE-PI3K pathway on the polarization of M1/M2 microglia. A.** Percentage of CD86 and CD206 positive cells detected by flow cytometry.** B.** The mRNA expression of M1 and M2 inflammatory factors. Compared with the OGD+PBS group, the M1 cytokines (IL-1β, iNOS and TNF-α) were significantly down-regulated, while the M2 cytokines (IL-4, IL-10 and Arg-1) were up-regulated. **C.** Expression of inflammatory factors in the supernatant detected by ELISA. After adding inhibitors, the concentration of IL-1β and TNF-α was significantly reduced, and the concentration of IL-10 increased. N=3 per group. * Represents statistical significance compared with the OGD+PBS group. * p<0.05, ** p<0.01, *** p<0.001.

Then, the polarization of M1/M2 microglia was detected. Flow cytometry showed that by inhibiting the HMGB1, RAGE, and PI3K/Akt pathways, the proportion of CD86+ cells decreased and that of CD206+ cells increased significantly (Fig. 5A). Also, qPCR exhibited that by inhibiting HMGB1, RAGE, and PI3K/Akt pathways, the mRNA levels of iNOS, IL-1β and TNF-α were significantly reduced, and the mRNA expression of IL-4, IL-10 and Arg-1 were varyingly upregulated (Fig. 5B). ELISA showed that the concentrations of IL-1β and TNF-α significantly reduced and the concentration of IL-10 increased through the inhibition of the HMGB1, RAGE, and PI3K/Akt pathways (Fig. 5C). The above results indicate that under OGD conditions, the inhibition of HMGB1/RAGE-PI3K hampered the polarization of microglia to the M1 phenotype and promoted polarization to the M2 phenotype, diminishing the expression of pro-inflammatory cytokines.

### 3.4 HMGB1-RAGE/PI3K Pathway Activated in Neonatal HIBD rat model

**Figure 6:**
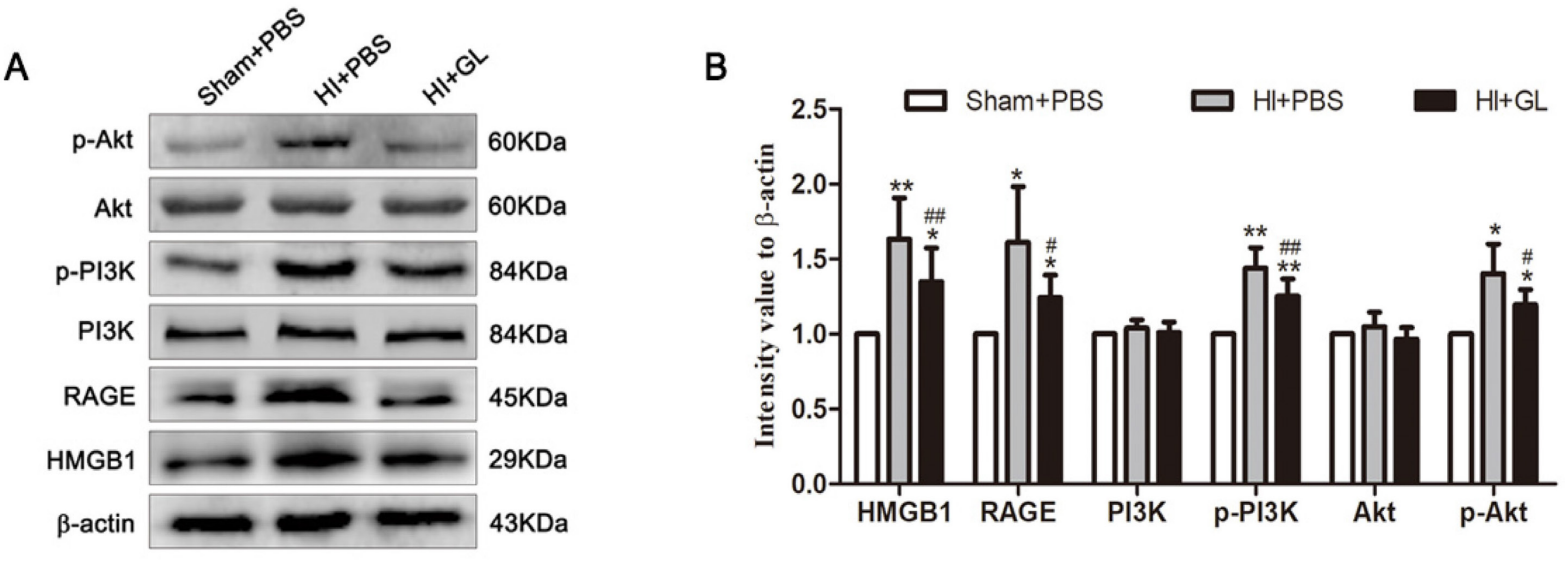
** The activation of HMGB1/RAGE-PI3K pathway in neonatal HIBD rat model. A.** The immunoblots of HMGB1, RAGE, PI3K/p-PI3K and Akt/p-Akt in neonatal rats 7d after HI. Compared with the Sham+PBS group, the expression of RAGE in the HI+PBS group was upregulated, and the phosphorylation of P85 subunit and S473 occurred in PI3K/Akt pathway. Pretreatment with GL partially neutralize these changes. **B.** Quantitative results of each protein. N=5 per group. * Represents statistical significance compared with the Sham+PBS group. * p<0.05, ** p<0.01. #Represents statistical significance compared with the HI+PBS group. # p<0.05, ## p<0.01.

In our previous study, we discovered that HMGB1 could aggravate brain damage in a neonatal HIBD model by regulating the polarization imbalance of microglia. In this study, the OGD model of microglia *in vitro* suggests that HMGB1 causes an imbalanced M1/M2 microglial polarization through the activation of the RAGE-PI3K/Akt pathway. The expression of the RAGE-PI3K/Akt pathway in the neonatal HIBD model was further evaluated. The results showed that compared with the Sham + PBS group, the expression of HMGB1 and RAGE were significantly upregulated, and the phosphorylation of PI3K and Akt was also activated 7 days after HI. Pretreatment with the HMGB1 inhibitor GL downregulated the expression of RAGE and weakened the phosphorylation of PI3K and Akt. Consistent with the *in vitro* results, these findings indicate that the RAGE-PI3K/Akt pathway was activated after HI exposure in the neonatal HIBD model, and phosphorylation and activation of RAGE-PI3K/Akt were partly dependent on HMGB1 (Fig. 6A and B).

### 3.5 Effect of Inhibiting HMGB1-RAGE/PI3K on HI-Induced Brain Injury

**Figure 7:**
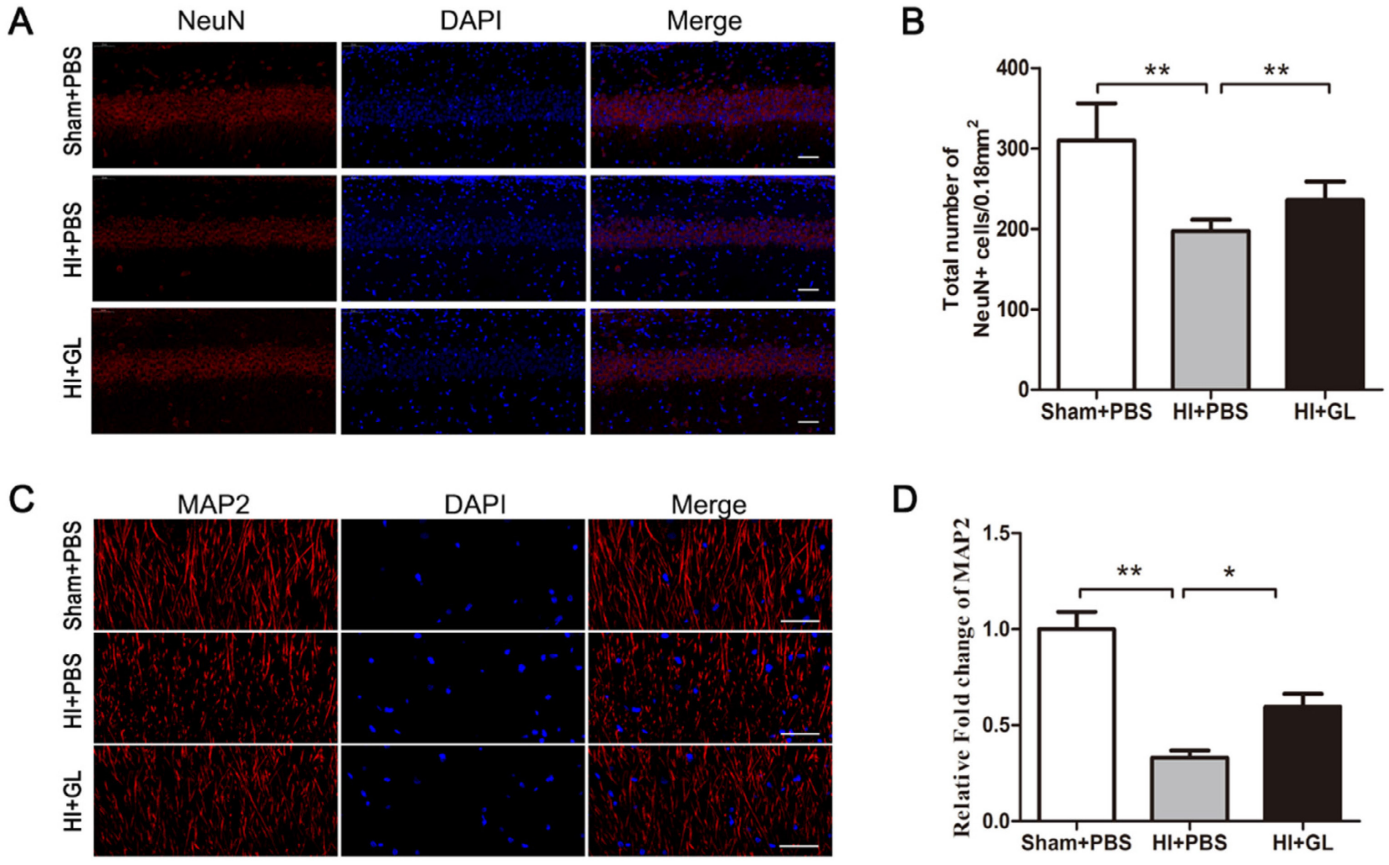
** The neuronal changes in neonatal HIBD rat model by inhibiting HMGB1/RAGE-PI3K pathway. A.** Immunofluorescent labeling of neurons (NeuN, red) with DAPI (blue) in the CA1 region 7d after HI. scale bar=50 μm. **B.** The statistical results of NeuN+ cell numbers in 0.18 mm^2^ area. The number of NeuN+ cells significantly decreased after HI and it had an increase in HI + GL group compared to HI + PBS group. N=5 for each group, bars represent the mean ± SD. ** p<0.01. **C.** Immunofluorescent labeling of neurites (MAP2, red) with DAPI (blue) in the CA1 region 7d after HI. scale bar=50 μm. **D.** Quantitative analyses of MAP2 fluorescence intensity (relative OD value). The intensity of MAP2 significantly decreased after HI and it had an increase in HI + GL group compared to HI + PBS group. N=5 for each group, bars represent the mean ± SD. * p<0.05, ** p<0.01.

Our previous study conveyed that HMGB1 aggravates microglial-induced neurotoxicity under OGD conditions. In this study, HMGB1 inhibitor GL was utilized and the activation of RAGE-PI3K/Akt pathway in HIBD rat model was alleviated (Fig. 6A and B). Then brain injury was evaluated by immunofluorescence. The outcomes highlighted that the number of NeuN-positive neurons in the CA1 area decreased significantly after HI insult, and inhibition of RAGE-PI3K/Akt pathway by pretreatment with GL partially reduced neuronal loss (Fig. 7A and B). Meanwhile, we used MAP2 as a marker to identify the changes of nerve dendrites and found that the density of nerve dendrites in CA1 also decreased after HI exposure. GL pretreatment partially increased the density of the dendrites (Fig. 7C and D). These results suggest that HI-induced brain injury could be partly alleviated by inhibiting HMGB1-RAGE-PI3K/Akt pathway.

## 4. Discussion

As a damage-associated protein, HMGB1 promotes the production of pro-inflammatory cytokines through microglia, thereby triggering neuroinflammation. Microglia can be classified into two main forms: M1-type (pro-inflammatory) and M2-type (anti-inflammatory) 17. M1 microglia promotes neuronal death via the expression of pro-inflammatory mediators, such as IL-6, TNF-α and iNOS. Conversely, M2 microglia facilitates tissue repair and support neuronal survival through the secretion of anti-inflammatory cytokines such as TGF-β, IL-10 and arginase-1 18, 19. Normally, there exists a delicate balance between pro-inflammatory M1 and anti-inflammatory M2 microglia 20. Under pathological conditions, there exists an imbalance in microglial M1/M2 polarization that promotes neuroinflammation and affects the survival of neurons 21. Our previous study showed that HMGB1 may generate an imbalanced M1/M2 microglial polarization and cause neuronal injury, but the mechanism is not clear. In this study, we further found that HMGB1 facilitated polarization of microglia through RAGE-PI3K/Akt pathway *in vivo* and *in vitro*. Inhibition of HMGB1-RAGE-PI3K/Akt significantly alleviated the neuronal damage in HIBD.

We first established an OGD model *in vitro* and changed the expression of HMGB1 through cell transfection to clarify the effect of HMGB1 on the polarization of M1/M2 microglia. The results showed that under OGD conditions, HMGB1 promoted microglial polarization toward the M1 type, promoting neuroinflammation. This result is in line with our previous study *in vivo*. Several researchers have proven that HMGB1 promotes neuroinflammation and causes brain damage in many neurological diseases, such as traumatic brain injury 2, Alzheimer's disease 22, experimental autoimmune encephalomyelitis 23, and ischemic stroke 24. Hence, HMGB1 is now a potential target during the treatment of various neurological diseases and the outcomes in this study will provide further theoretical support.

RAGE is one of the main receptors of HMGB1 4. Studies show that after HI exposure, the expression of RAGE in neurons and non-neuronal cells is upregulated to varying degrees 25, 26. Although it was found that HMGB1-RAGE does not aggravate neuronal inflammation 27, others demonstrated that the activation of the HMGB1-RAGE axis could promote the neuroinflammatory response. In an animal model of ischemic stroke, RAGE expressed on microglia can mediate HMGB1 neurotoxicity. Bone marrow-derived cells transplanted into the brain after RAGE knockout effectively reduced the area of cerebral infarction 6. A recent study signified that the activation of HMGB1-RAGE could promote inflammation in microglia, and HMGB1 knockdown can inhibit the expression of RAGE and reduce neuroinflammation 7. *In vitro* experiments have shown that partial inhibition of RAGE expression can reduce neurotoxicity under OGD conditions 25, 26. We determined that the expression of RAGE was positively correlated with the expression of HMGB1, and HMGB1-RAGE could facilitate the polarization of microglia to M1 type and impede the polarization of microglia to the M2 type, resulting in the imbalanced polarization of M1/M2 microglia under OGD conditions. In the neonatal HIBD model, RAGE was upregulated, and hippocampal neurons were significantly injured. Pretreatment with GL inhibited RAGE expression and alleviated neuronal damage. Based on these studies, inhibiting the expression or activation of HMGB1-RAGE may alleviate the polarization of microglia to the M1 type, thereby reducing neuroinflammation.

The PI3K/Akt pathway regulates cell activities, including proliferation, apoptosis, and metabolism, and plays a central role in health and disease 9. Activated Akt is vital for initiating immune responses, while unchecked activation of this pathway has detrimental effects. The dysregulation of the PI3K/Akt signaling pathway is involved in pathogenesis of several nervous system diseases 27. As many of the key regulatory receptors that activate PI3K/Akt pathway are expressed by microglia, PI3K/Akt signaling in the brain appears to be intimately linked with microglial activity and activation 28. Previous studies found that the PI3K/Akt signaling pathway is involved in HI brain damage. Targeting the PI3K/Akt pathway could promote Akt phosphorylation, inhibit the expression of the apoptosis-related factor BCl2, and reduce neuronal apoptosis 29. Another study by Bhat and colleagues found that use of an anti-inflammatory modulator that promoted Akt activity in an experimental traumatic brain injury model resulted in decreased pro-inflammatory microglial activation and favorable outcomes in rodents 30. These results suggest that PI3K/Akt signaling may promote an anti-inflammatory response in microglia. On the contrary, other studies have found different results. Extrinsic stimuli with LPS in rodents leads to the activation of PI3K/Akt signaling in the CNS, which induces activation of microglial populations in the brain and is associated with exacerbated release of pro-inflammatory factors 31, 32. And dihydroartemisinin ameliorates LPS-induced neuroinflammation by inhibiting the PI3K/Akt pathway 33. Furthermore, a recent study showed that TLR4 promoted microglial pyroptosis by activating PI3K/Akt pathway after spinal cord injury 34. These results indicated that the activation of PI3K/Akt signaling could also promote neuroinflammation through microglia activation. In this study, we found that the PI3K/Akt pathway in HAPI microglia was activated under OGD conditions, encouraging microglial polarization towards the M1 type and favoring the expression of pro-inflammatory cytokines. Further analysis revealed that HMGB1-RAGE is located upstream of PI3K/Akt. The subsequent HIBD rat model suggested that neuronal damage after HI was related to the activation of the HMGB1-RAGE-PI3K/Akt pathway. These findings suggest that HMGB1 could activate the RAGE-PI3K/Akt signaling pathway under OGD conditions, leading to an imbalance in M1/M2 microglial polarization and neuroinflammation.

There are some limitations of our study. Firstly, as different pathological changes occur at different time points after HI 35, the results may be different after HI exposure at different time points. Unfortunately, in our study, we only detected only one time point (7d) after HI. In addition, we only applied the inhibitor GL to suppress the expression of HMGB1 in HIBD rat model, but did not reduce the expression of HMGB1 by gene knockout, which may have affected the results. Finally, the assessment of behavior changes is important in the study of brain injury. This study only detected morphological and quantitative changes in hippocampal neurons, but not the behavioral changes of neonatal rats. The aforementioned points therefore need to be addressed in further research.

## 5. Conclusion

Our findings suggest that HMGB1 contributes to the imbalance of M1/M2 microglial polarization through the RAGE-PI3K/Akt pathway under OGD conditions. Targeting HMGB1-RAGE-PI3K/Akt may assist in reducing neuroinflammation in HIBD.
